# Challenges to the infection control team during coronavirus disease 2019 (COVID-19) pandemic in a quaternary-care medical center in Saudi Arabia

**DOI:** 10.1017/ice.2021.72

**Published:** 2021-02-19

**Authors:** Hala A. Amer, Ibrahim A. Alowidah, Chasteffi Bugtai, Barbara M. Soule, Ziad A. Memish

**Affiliations:** 1 Prevention and Control of Infection Administration, King Saud Medical City, Riyadh, Saudi Arabia; 2 Community Medicine Research Department, National Research Center, Cairo, Egypt; 3 Health Services, King Saud Medical City, Riyadh, Saudi Arabia; 4 Joint Commission Resources/Joint Commission International, Six Sigma Yellow Belt, Chicago, Illinois, United States; 5 Research and Innovation Center, King Saud Medical City & College of Medicine, Al Faisal University, Riyadh, Saudi Arabia; 6 Hubert Department of Global Health, Rollins School of Public Health, Emory University, Atlanta, Georgia

## Abstract

**Background:**

King Saud Medical City (KSMC) is a quaternary care center based in the center of the capital city, Riyadh, Kingdom of Saudi Arabia (KSA), and it is one of the key Ministry of Health (MoH) facilities dedicated to the care of coronavirus disease 2019 (COVID-19) patients in the central region.

**Methods:**

A comprehensive surge plan was promptly launched in mid-March 2020 to address the pandemic, and it expanded in a phase-wise approach. Supporting the capacity of the infection prevention and control department (IPCD) was a main pillar of the surge plan. Task force infection control teams were formed to tackle the different aspects of pandemic containment processes. The challenges and measures undertaken by the IPC team are described here.

**Conclusion:**

Infection prevention and control staff are frontline responders in public health emergencies like COVID-19, and a solid infection prevention and control system in the healthcare setting supported by qualified and sufficient manpower, a well-developed multidisciplinary team approach, electronic infrastructure, and efficient supply utilization are required for effective crisis management.

The first case of coronavirus disease 2019 (COVID-19) in the Kingdom of Saudi Arabia (KSA) was confirmed on March 2, 2020, and by December 2020, 362,741 people had been infected there.^1^ For healthcare facilities, the preparedness effort has focused on early detection of cases and prompt isolation of suspected or confirmed cases, managing supply shortages, retraining all healthcare workers (HCWs) on proper donning and doffing of personal protective equipment (PPE), and implementing effective infection prevention and control (IPC) measures. These key strategies were undertaken to mitigate the impact of the pandemic on the frontline healthcare providers and on the health system as a whole.^2,3^

King Saud Medical City (KSMC), a 1,700-bed advanced quaternary care center comprises 3 major hospitals (general, pediatrics, and maternity) as well as dental and dialysis centers.   KSMC’s core competencies are emergency care, trauma, orthopedics, burn, bariatric surgery, and critical care. KSMC is based in the center of the capital city, Riyadh, Kingdom of Saudi Arabia (KSA) and is one of the key Ministry of Health (MoH) facilities dedicated to the care of COVID-19 patients in the central region. A comprehensive surge plan was launched in mid-February 2020 to address the pandemic, then it expanded in a phase-wise approach in response to the expected volume of suspected or confirmed COVID-19 cases to be admitted to the hospital.

The overall isolation capacity increased gradually from 28 to 82 single isolation beds (reaching almost triple its original census) in addition to allocation of cohorting wards for confirmed cases. During the peak of the COVID-19 curve in the KSA, which occurred in mid-June 2020, the average occupancy of confirmed COVID-19 cases in cohorting areas was ∼320 patients, and 50% required critical care (Fig. 1).

**Fig. 1.**
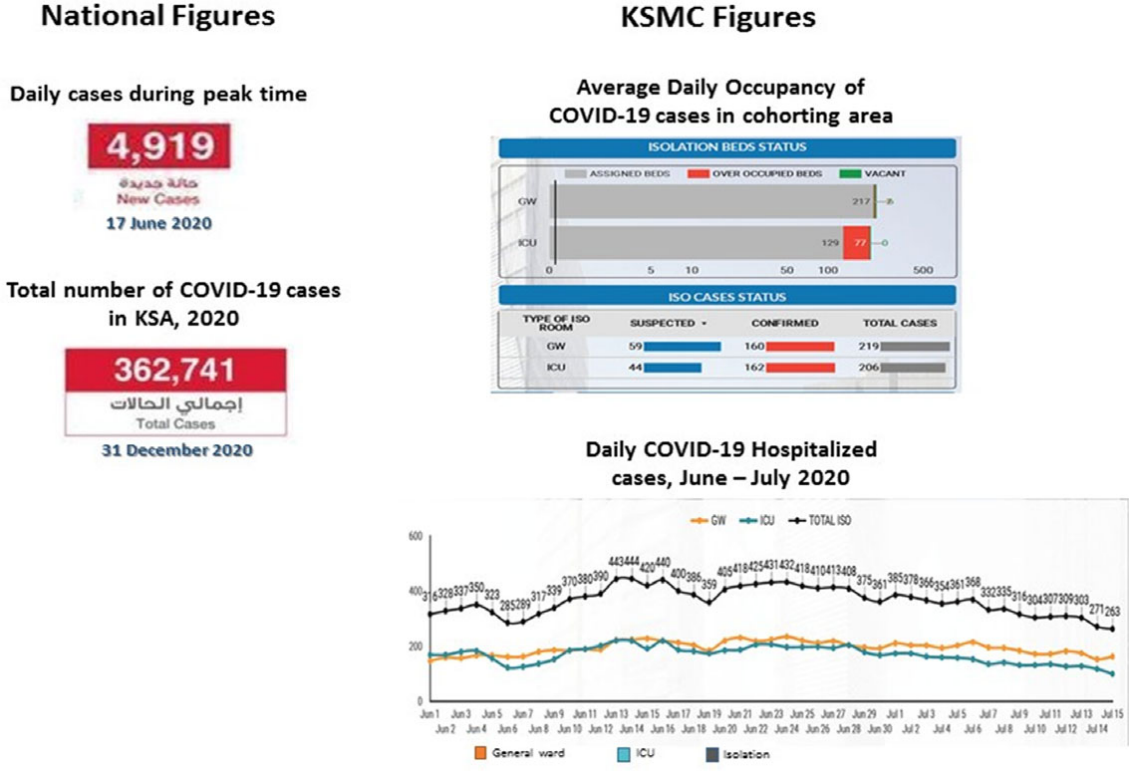
COVID-9 statistics: number of reported cases in Saudi Arabia isolation occupancy at King Saud Medical City.

Supporting the capacity of the infection prevention and control department (IPCD) was one of the main pillars of the KSMC pandemic surge plan. Infection prevention and control staff are frontline responders in public health emergencies like the COVID-19 pandemic. We considered a disaster management approach focusing on team structure to provide care and ongoing communication while managing risk and reprioritizing activities. The work dynamic of the infection control team was reconfigured to cope with the crisis. Herein, we describe the challenges and measures undertaken by the IPC team in responding to the COVID-19 pandemic.

## Methods

In mid-February 2020, as an early response to the global COVID-19 situation and aligned with our institutional disaster management plan, the IPCD at KSMC called for the formation of a multidisciplinary outbreak committee headed by the chief medical officer and including representatives of key stakeholders from nursing, emergency, intensive care, infectious diseases, respiratory therapy, patient affairs, laboratory, engineering affairs, medical supply, public relations, and information technology departments. The committee met daily to continually review the pandemic curve globally and locally.

### Virus prevention and mitigation strategies

#### Patient pathway

**Fig. 2.**
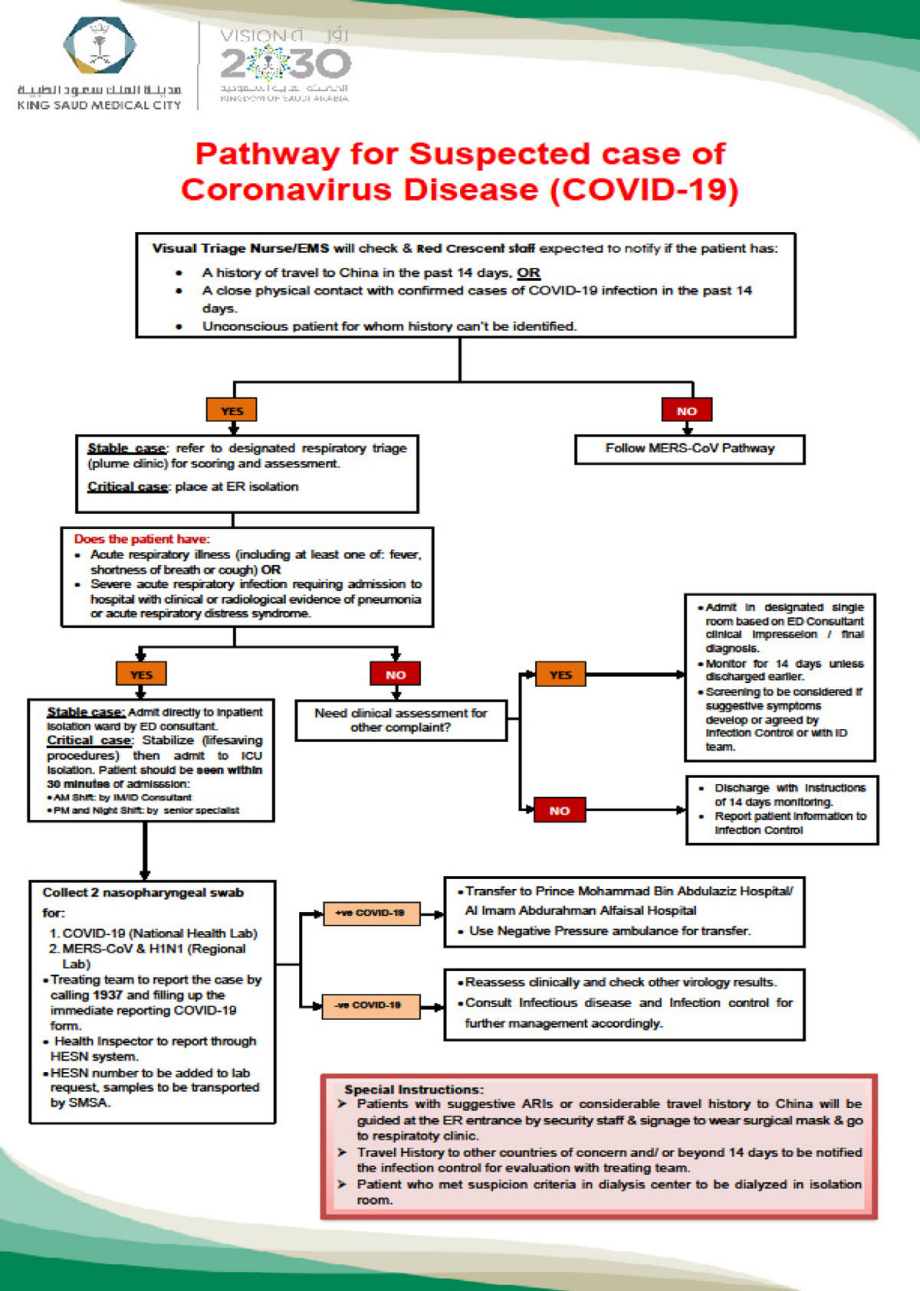
Pathway of suspected and confirmed cases of COVID-19.

A patient pathway was developed starting from the emergency department (ED) to admission or discharge to provide the most efficient and safe care and to limit unnecessary exposure to HCWs (Fig. 2).

#### Crowd control

Predetermined decisions were applied to control the in-hospital crowd. For patients and their families, crowd control included halting all elective services, restricting visitors, and installing temperature screening stations at building entrances. For HCWs, the hospital initiated periodic rotation of employees to alternative teams to maintain physical distancing in administrative and clinical departments. These measures aimed to minimize the risk of COVID-19 transmission among staff and to maintain the manpower pool. Also, the institution’s electronic portal and communication system were modified to be accessible to allow work from home, making all meetings and academic events virtual.

#### Rapid response team

In addition, any newly discovered case of COVID-19 within the hospital premises activated the rapid response team, which was trained to take required actions including contact tracing, quarantine, and management of an influx of infectious cases.

### Task force teams

**Fig. 3.**
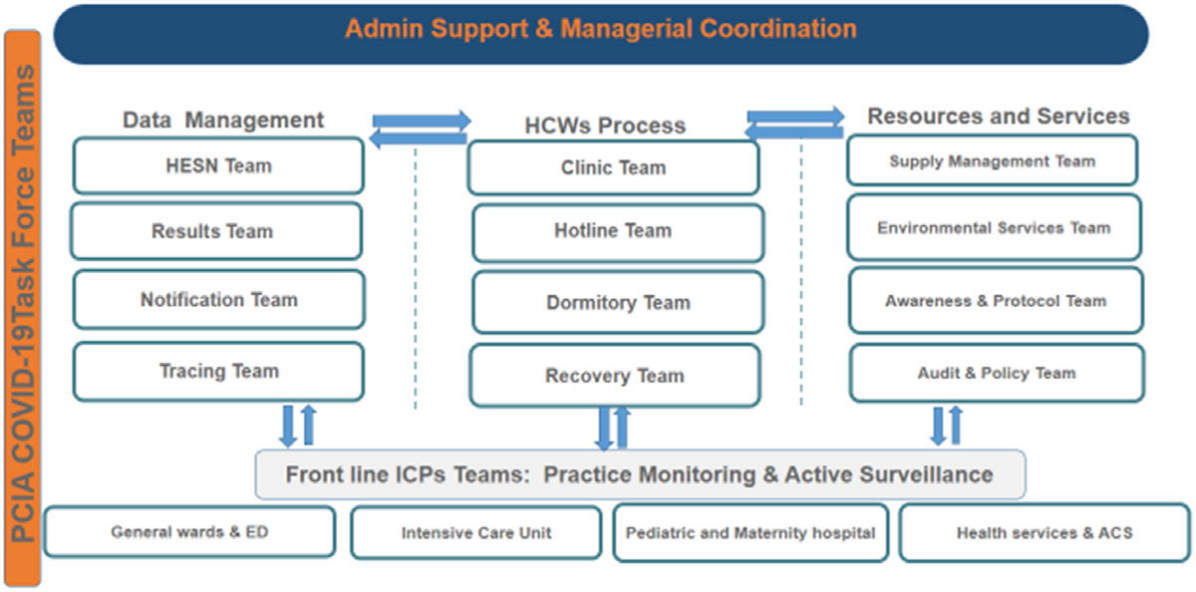
COVID-19 governance and collaboration task force teams. Note. PCIA, Prevention and Control of Infection Administration; ICPs, infection control practitioners.

With the first reported COVID-19 case in the KSA, multiple task force teams were created by the IPCD to manage various essential operational functions (Fig. 3). The work dynamic of those teams was coordinated with oversight at the managerial level. Because it was beyond the capacity of the existent IPC staff to operate such emergency services, volunteer opportunities were announced to support the IPC team, which doubled the IPCD manpower from 30 to 60 HCWs. Volunteers from departments and programs that were downsized or those that held their services, such as the dental department and internship and academic programs, were recruited and trained to fulfill the required duties.

The task force teams were organized into 4 main groups:

1. Surveillance and data management group. This group ensured that the flow of data and information would proceed in a smooth, timely, and effective manner among all groups as needed (Fig. 4).
  **Fig. 4.**
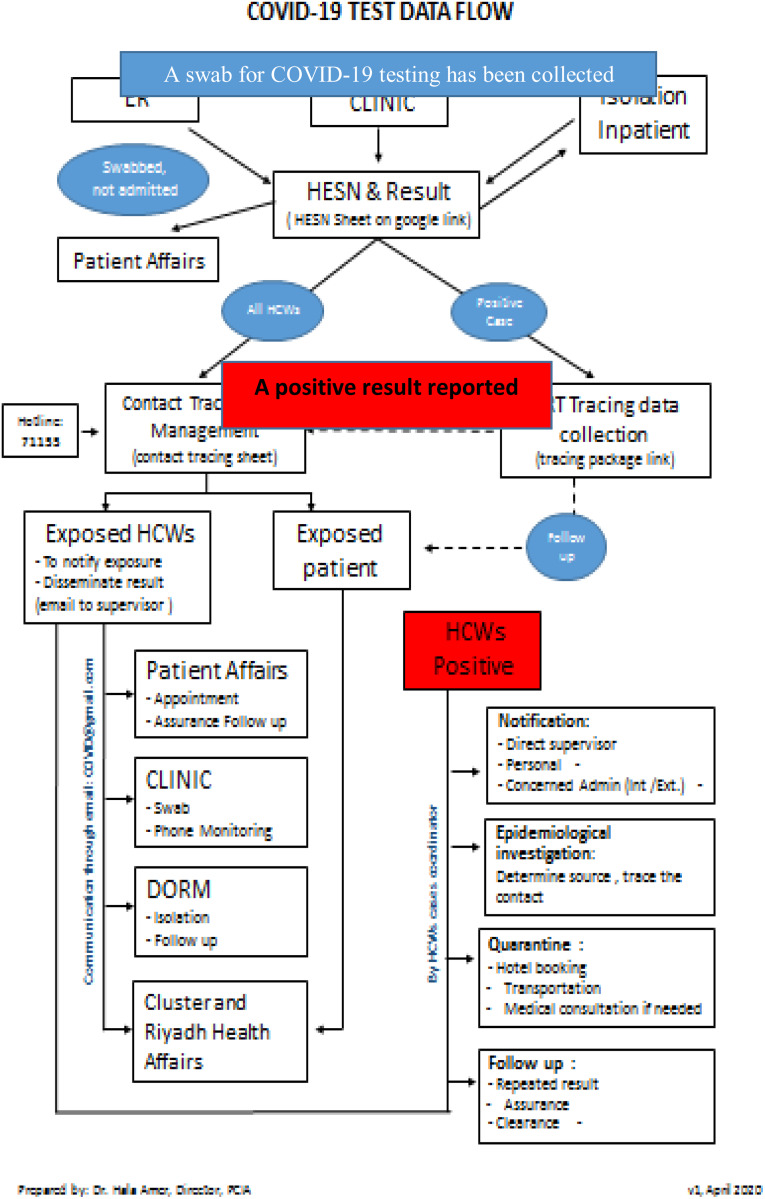
COVID-19 test data flow.
2. Registration team. As a national mandate and in compliance with the Saudi MoH reportable diseases notification process, any person tested for COVID-19 was registered in a national database called the Healthcare Electronic Surveillance Network (HESN). A team of public health inspectors and volunteers from the medical staff were trained to access the system and to enter required data.
3. Results notification team. The results team tracked the results of registered COVID-19 test samples and entering them on a Google spread sheet that was accessible to all clinical areas. An algorithm for triaging suspected cases and testing for COVID-19 was developed based on a surveillance case definition and triaging system approved by the Saudi Center for Disease Control (Fig. 2).^4^ Clinical specimens for COVID-19 screening (including nasopharyngeal swabs) were initially sent to a centralized national laboratory for polymerase chain reaction assay (PCR) testing. Later, testing was decentralized to many regional laboratories to increase testing capacity and to reduce turnaround time from 48 hours to 12 hours.

Driven by the royal order to offer COVID-19 testing and management as an unpaid service for all citizens and expatriates, accessibility to the services expanded. The number of COVID PCR tests increased from 10,000 tests per day to >100,000 tests daily. At KSMC, 41,916 COVID-19 samples were collected between mid-January and the end of December 2020.

1. Notification and tracing teams. Proper contact tracing is an essential element in the containment of any infectious disease outbreak. Once a positive COVID-19 result was reported to the hospital, an e-mail was sent by the notification team to the concerned department(s). In parallel, the contact-tracing team tracked the pathway of hospitalized positive cases and interviews the nonhospitalized cases by telephone to identify close contacts among HCWs and patients. By the end of December, 4,914 positive cases had been traced. In addition, the notification team also communicated negative results by telephone.

**Fig. 5.**
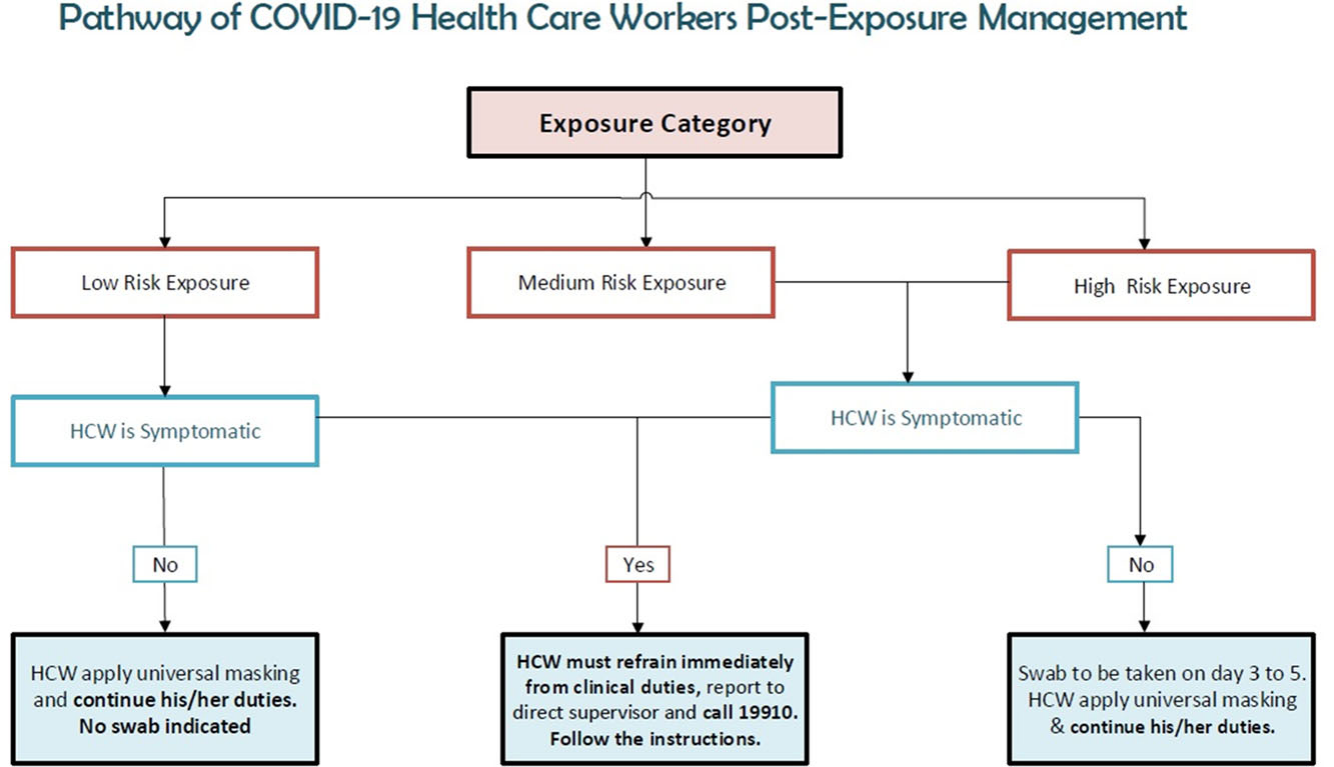
COVID-19 healthcare worker management after exposure.

Exposed personnel were also notified about their risk of exposure in order to apply the postesxposure protocol according to approved guidelines (Fig. 5). Exposure in the community (eg, household and workmates contacts) was traced and managed by the public health authority.

### HCW postexposure management group

A special process was established for addressing HCW COVID-19 concerns. The HCW postexposure management group worked on this matter using 4 established subteams.

1. Clinic team. In the beginning of the pandemic, to facilitate testing for COVID-19 and to spare the limited negative-pressure rooms, our negative-pressure ambulance was used by the clinic team to collect COVID-19 test swabs from hospital employees. The main indication for the swab test was returning from international travel.

As the situation evolved and local transmission incidents increased, a designated COVID-19 employee clinic with a broader scope of service involving family medicine physicians and internists was allocated for personnel testing in a well-ventilated noncrowded location near one of the hospital gates. This clinic serves HCWs returning from abroad, who were exposed to a positive case, and/or who presented with COVID-19 symptoms. The clinic processes aligned with national protocol for HCWs.

1. Hotline team. Virtual consultation was accessible to respond to employee concerns via a hotline.
2. The dormitory team. Uniquely in the KSA, the healthcare system depends on many expatriates who mostly live in a common housing building sponsored by the hospital. At KSMC, >2,000 female staff, mainly nurses, live in 3 housing buildings with ∼10 persons in each flat sharing the same kitchen and 2 bathrooms. One of those buildings has been designated for isolation of staff living in the dormitory whenever quarantine is indicated. A team was created to oversee the dormitory isolation facility, to initiate and discontinue isolation of staff living in the dormitory, to check isolated staff and meet their needs, and to ensure adherence of all staff in the dormitory to all the necessary preventive measures.

Continuous awareness and monitoring inside the dorm was conducted to minimize staff interaction and exposure between different flats by preventing social gathering in common lobbies. However, it was difficult to control the interactions inside each flat. Monitoring continued in order to detect and isolate any dormitory resident presenting with COVID-19 symptoms.

1. Recovery team. A recovery team was assigned to guarantee proper and timely follow-up of HCWs affected by COVID-19 using the established return-to-work process and to ensure that manpower capacity was maintained to avoid staff shortages. Early in the pandemic, the KSA MOH regulation mandated that any positive case (including HCWs) either be admitted to the hospital or isolated in a designated quarantine hotel. Two consecutive negative PCR tests were required for release from isolation. In some extreme cases, this process took ∼6 weeks and required extended follow-up by the team. With guideline updates, home isolation was allowed if convenient, and positive HCWs were allowed to resume duty 10 days after their positive result date with symptom resolution.

### Resources and services group

The resources and services group was responsible for support services and resources management and was composed of 4 subteams.

1. Supply management team. The shortage of essential medical supplies was one of the major challenges faced by the healthcare system during the COVID-19 pandemic. Critical shortages were identified for PPE (mainly N95 respirators) and disinfection supplies. The supply management team was assigned to work closely with supply-chain stakeholders to oversee the inventory of critical PPE and other supplies, to calculate need and consumption, to evaluate new products, and to request the purchase of equipment and consumables required for infection control practices and COVID-19 prevention. A database was created that included items defined by the MoH as essential infection control supplies. The strategies implemented by this team were successful in optimizing PPE distribution and utilization.

New products were evaluated by IPCD and introduced by the team such as transportation capsules, HEPA filters, ultraviolet disinfection machine and powered air purifying respirators (PAPRs).

1. Environmental services team. The healthcare environment and fomites can be contaminated with SARS-CoV-2, leading to possible healthcare-associated transmission to HCWs or patients.^5^ It is crucial to apply proper environmental disinfection to minimize risk of indirect transmission of COVID-19 infection. The environmental health team determined cleaning and disinfection protocols for patients’ rooms, utility rooms, nursing stations, offices, elevators, employee transportation buses, patient transportation capsule, public areas (eg, hospital garden and coffee shops). Selection of cleaning and disinfection products was based on approved effectiveness and compatibility according to national and international guidelines.^6^

Hydrogen peroxide (H_2_O_2_) fogging and ultraviolet light (UV) technology were used for the terminal cleaning of isolation rooms after discharge of positives cases as well as for scheduled mass cleaning of all rooms in certain hospital units. Impregnated quaternary ammonium wipes and chlorine spray were used for routine cleaning as appropriate. This team worked closely with the supportive services team conducting daily rounds to observe cleaning quality and other practices such as transportation of linen and medical waste. The frequency of waste collection was increased to meet the extra demand. To overcome the challenges of language barriers and lack of competency among poorly educated housekeepers, training was delivered to them using posters, videos, and practical demonstrations.

1. Awareness and protocol team. Given the nature of this pandemic and the continuous changes in guidelines and protocols, the awareness team was tasked with keeping all HCWs updated “right on time” with current infection prevention and control information. Face-to-face education sessions and practical bedside training to address staff concerns were performed in the early stages of the pandemic, and these were soon replaced by remote education techniques. An e-learning certified course with competency assessment was launched for HCWs, especially those assigned to critical care units, emergency departments, and isolation units. Additionally, many virtual sessions were presented regarding specific topics such as the use of PAPR and protocols for PPE reuse and extended use.

Messages were circulated about preventive measures through the hospital portal. Other tools for education included messages displayed on computer screens and reminder posters located throughout the hospital, guiding HCW behaviors and providing information for the public when visiting was resumed with restricted numbers and shorter duration (after the pandemic curve flattened).

1. Audit and policies team. The Central Board of Accreditation of Healthcare Institutions (CBAHI) a national accreditation body in Saudi Arabia (similar to The Joint Commission in the United States) issued a checklist consisting of 7 main standards for assessing healthcare preparedness for MERS-CoV and COVID-19.^7^ Implementation of those standards was audited through scheduled and unscheduled leadership tours, which enhanced interaction with frontline staff, ensured compliance with the standards, and addressed personnel concerns. This team also worked closely with the quality management department to develop COVID-19 prevention policies including both the generic hospital-wide policies and specific services policies, such as dealing with COVID-19 in the operating room.

### Frontline ICP group

This group consisted of 4 teams and covered different hospital units. Each team worked on enhancing the implementation of infection prevention and control measures through continuous monitoring of staff practices, immediate feedback and awareness, and checking the adequate distribution of required resources. Hand hygiene compliance was also monitored regularly by the teams using the World Health Organization observation form.^8^

A simplified monitoring tool for COVID-19 was created for the hospital including all preventive measures such acute respiratory illness monitoring for HCWs, social distancing, universal masking, hand hygiene, PPE practices, and environmental and equipment cleaning. The form also included a category for reporting of malpractices to the concerned supervisor, employing engineering parameters and a room-entry log book in isolation rooms (Appendix 1 online). The form was completed by infection control practitioners on all rounds that occur daily on the morning shift in all hospital units and are repeated on the evening shift in special care units including emergency department, critical care unit, and isolation wards.

Frontline infection control teams also consulted with treating teams to increase proper utilization of the isolation ward beds. This occurred through continuous joint rounds and shared decision making about discontinuing isolation and discharging suspected or confirmed COVID-19 cases.

## Discussion

According to the national preparedness plan for emerging infectious diseases, a series of proactive infection control measures were activated by the regulatory and governing bodies of the KSMC. The key interventions included a bundle of IPC measures for early recognition, isolation, notification, and molecular diagnostic testing for all suspected COVID-19 cases.^8^ Building on the MERS-CoV experience, the healthcare system in the KSA was partially prepared for the COVID-19 pandemic.^9^

Forming competent well-structured task force teams at the institutions was a successful approach to dealing with the crisis. We established and monitored the strategies implemented in the bundle of measures, and we exceeded them according to our situational analysis. Collaboration and partnership between the infection control teams and other services and departments was crucial to these efforts. Continuous communication between the teams through daily virtual meeting and many WhatsApp groups supported the coordination and was a key factor of our success.

There is always room for improvement in overcoming new and continuing challenges. The main obstacles faced by the teams included technical concerns uploading data into the national surveillance software (HESN) because it was not integrated with most hospital health information systems (HISs) and had to be entered manually. The timing of receiving the results of COVID-19 tests from the laboratory was inconsistent, which was a challenge for the concerned teams who continued tracking the results on a 24/7 basis. Delayed turnaround times delayed important decisions and actions such as releasing cases from isolation, performing indicated diagnostic and therapeutic procedures, and returning HCWs to duty. Lack of an easily accessible registry of employee information and electronic medical records of patients were drawbacks that affected accuracy and timing of the containment procedures and increased the workloads for all teams.

Despite these challenges, improvements were made as the pandemic evolved. An agreement was reached with the laboratory to dedicate sample receiving times and to upload the results within a shorter, unified turnaround time. Initiatives for building databases of all required information were started. Many electronic healthcare applications have been developed worldwide, and many have been launched by the public health system in the KSA to combat this pandemic. These applications support strategies such as population screening, notification of COVID-19 test results, and advice about IPC practices. Those applications include *Sehhaty* (my health), *Tatamman* (rest assured), *Takasi* (tracing), *Tawakkalna* (COVID-19 KSA official app), and *Tabaud* (keep distance).^10^

Artificial intelligence helps combat the virus by tracking its spread, identifying high-risk patients, monitoring treatment, and controlling infection in real time.^11,12^ Using digital approaches helps HCWs overcome manage high workloads arising from a sudden and massive increase in the number of very ill patients. Moreover, artificial intelligence can help to predict mortality risk by adequately analyzing the previous data of the patients.^13–15^

Rising HCW anxiety and fear was a major challenge that resulted in high inflow at COVID-19 screening clinics to rule out COVID-19 infection. This anxiety has been managed by virtual clinic and hotline services for consultation, assurance related to possible exposures, and a planned appointment system. Staff anxiety also resulted in overuse of PPE, which affected the supply stock.

Breaking the news of a positive COVID-19 result to the infected HCWs required a well-trained tracing team. The notification was acknowledged by a variety of reactions depending on the infected HCW’s perception about their COVID-19 prognosis. A mental health clinic was launched to manage the psychological consequences of COVID-19 fear and infection among HCWs. The significant psychological impact of the COVID-19 pandemic on frontline staff has been described in many recent studies.^16,17^ Being overwhelmed, dealing with shortages of medical equipment, concerns about infecting family members, individual coping styles and culture, in addition to surrounding support systems are important factors affecting HCW stress levels and responses to trauma. In the hospital, many channels have been created as a proactive approach to manage employee stress levels and to prevent burnout, such as weekly nursing virtual forums and a periodic newsletter series entitled “Because We Care” for counseling, peer discussion, and sharing experiences and life stories related to the pandemic (Appendix 2 online). The COVID-19 pandemic presents challenges and opportunities for infection prevention and control practices. Despite an increase in HCW compliance with best practices, the pandemic has interrupted the routine surveillance system and the regular reporting of healthcare-associated infections (HAIs) and other key performance measures and initiatives. Significant diversion of infection prevention resources has been required to help manage the outbreak in the hospital and at the health-system level.^18^

In summary, a solid infection prevention and control system in the healthcare setting supported by qualified and sufficient manpower, a well-developed multidisciplinary team approach, electronic infrastructure and efficient supply utilization are required for effective crisis management. In all aspects, the IPC system should be aligned and integrated with national public health plans and organizational missions and policies.

## Supplementary material

For supplementary material accompanying this paper visit http://dx.doi.org/10.1017/ice.2021.72. click here to view supplementary material
