# Supplementary material for: Challenges to the infection control team during coronavirus disease 2019 (COVID-19) pandemic in a quaternary-care medical center in Saudi Arabia
Source: Infect Control Hosp Epidemiol. 2021 Feb 19:1–8. doi: 10.1017/ice.2021.72 (PMC8458846; doi:10.1017/ice.2021.72)
Supplement: Supplementary file 1 [file icesup.zip › S0899823X21000726sup001.docx]

Infection Control E. News:
