# Supplementary material for: Challenges to the infection control team during coronavirus disease 2019 (COVID-19) pandemic in a quaternary-care medical center in Saudi Arabia
Source: Infect Control Hosp Epidemiol. 2021 Feb 19:1–8. doi: 10.1017/ice.2021.72 (PMC8458846; doi:10.1017/ice.2021.72)
Supplement: Supplementary file 1 [file icesup.zip › S0899823X21000726sup002.docx]

| IPC staff Name: | | Floor/Area: |
| --- | --- | --- |
| Week: From: To: | | Shift : |
| PRACTICES | COMMENT | ACTION TAKEN/ RECOMMENDATION |
| ARIs* SCORE for HCWs and PATIENTS |  |  |
| SOCIAL DISTANCING |  |  |
| UNIVERSAL MASKING |  |  |
| HAND HYGIENE |  |  |
| PPE USE |  |  |
| EQUIPMENT CLEANING |  |  |
| ENVIROMENT CLEANING & LINEN |  |  |
| WASTE MANGMENT |  |  |
| No. of REPORTED MALPRACTICE |  |  |
| ISOLATION ROOMS:  Eng. Parameters  Logbook & Signage |  |  |
| General Notes: | | |

ARIs*: Acute Respiratory Illness.

One form for each area/ floor per week. To be discussed daily with your coordinator and follow up daily with Head Nurse
